# COVID-19 e Injúria Miocárdica em UTI Brasileira: Alta Incidência e Maior Risco de Mortalidade Intra-Hospitalar

**DOI:** 10.36660/abc.20200671

**Published:** 2021-01-13

**Authors:** Jorge Henrique Paiter Nascimento, Rafael Lessa da Costa, Luiz Fernando Nogueira Simvoulidis, João Carlos de Pinho, Roberta Santos Pereira, Andrea Dornelles Porto, Eduardo Costa de Freiras Silva, Liszt Palmeira Oliveira, Max Rogerio Freitas Ramos, Gláucia Maria Moraes de Oliveira

**Affiliations:** 1 Universidade Federal do Rio de Janeiro Rio de JaneiroRJ Brasil Universidade Federal do Rio de Janeiro, Rio de Janeiro, RJ - Brasil; 2 Hospital Unimed-Rio Rio de JaneiroRJ Brasil Hospital Unimed-Rio, Rio de Janeiro, RJ - Brasil

**Keywords:** COVID-19, Coronaviírus, Betacoronavírus, SARS – CoV-2, Infecção, Miocardite, Infarto do Miocárdio, Hospitalização, Morbidade

## Abstract

**Fundamentos:**

A incidência de injúria miocárdica (IM) em pacientes com COVID-19 no Brasil é pouco conhecida e o impacto prognóstico da IM, mal elucidado.

**Objetivos:**

Descrever a incidência de IM em pacientes com COVID-19 em unidade de terapia intensiva (UTI) e identificar variáveis associadas à sua ocorrência. O objetivo secundário foi avaliar a troponina I ultrassensível (US) como preditor de mortalidade intra-hospitalar.

**Métodos:**

Estudo observacional, retrospectivo, entre março e abril de 2020, com casos confirmados de COVID-19 internados em UTI. Variáveis numéricas foram comparadas com teste t de Student ou U de Mann-Whitney, sendo o teste X2 empregado para as categóricas. Realizou-se análise multivariada com as variáveis associadas à IM e p<0,2 objetivando determinar preditores de IM. Curva ROC foi empregada para determinar o valor da troponina capaz de predizer maior mortalidade intra-hospitalar. Funções de sobrevida foram estimadas pelo método de Kaplan-Meier a partir do ponto de corte apontado pela curva ROC.

**Resultados:**

Este estudo avaliou 61 pacientes (63,9% do sexo masculino, média de idade de 66,1±15,5 anos). A IM esteve presente em 36% dos casos. Hipertensão arterial sistêmica (HAS) [RC 1,198; IC95%: 2,246-37,665] e índice de massa corporal (IMC) [RC 1,143; IC95%: 1,013-1,289] foram preditores independentes de risco. Troponina I US >48,3 ng/ml, valor determinado pela curva ROC, prediz maior mortalidade intra-hospitalar [AUC 0,786; p<0,05]. A sobrevida no grupo com troponina I US >48,3 ng/ml foi inferior à do grupo com valores ≤48,3 ng/dl [20,3 x 43,5 dias, respectivamente; p<0,05].

**Conclusão:**

Observou-se alta incidência de IM na COVID-19 grave com impacto em maior mortalidade intra-hospitalar. HAS e IMC foram preditores independentes de risco de sua ocorrência. (Arq Bras Cardiol. 2020; [online].ahead print, PP.0-0)

## Introdução

A COVID-19, nomeada conforme orientação da Organização Mundial da Saúde (OMS), é causada pelo novo coronavírus sob a designação de SARS-CoV-2 ( *severe acute respiratory syndrome coronavirus 2* ) e teve seu surto descrito pela primeira vez na cidade de Wuhan, localizada na China, ao final de 2019. Foi declarada como emergência em saúde pública de interesse internacional em 30 de janeiro de 2020 e, na ocasião em que este estudo foi escrito, existiam cerca de 12.964.809 casos confirmados e 570.288 óbitos em todo o mundo.^1^ No Brasil, até o dia 14 de julho de 2020, foram confirmados 1.926.824 casos com 74.133 óbitos.^2^

A maioria dos casos de infecção por SARS-CoV-2 não é grave, incluindo apresentações assintomáticas ou oligossintomáticas. Todavia, relatos sugerem que até 20% dos indivíduos infectados requerem hospitalização, e desses, até 25% necessitam de cuidados em unidade de terapia intensiva (UTI).^3 , 4^ Essas taxas variam de acordo com diferenças culturais em relação aos critérios de admissão à UTI e características regionais, como idade da população e prevalência de outras comorbidades. Desenvolvimento de dispneia e síndrome respiratória aguda grave são as indicações mais comuns de internação em UTI.^3 - 5^

O acometimento cardíaco de pacientes em estado crítico por COVID-19 não é incomum e abrange uma grande variedade de apresentações como arritmias, cardiomiopatias e injúria miocárdica.^5 - 7^ A incidência de injúria miocárdica em pacientes hospitalizados varia entre 7% e 28% e algumas fontes sugerem correlação com piores desfechos clínicos.^7 - 9^ No entanto, as causas de lesão miocárdica e sua contribuição prognóstica ainda não foram bem elucidadas.

Este trabalho teve por objetivo primário descrever a incidência de injúria miocárdica em pacientes internados por COVID-19 em UTI e identificar possíveis fatores de risco relacionados à sua ocorrência. Nosso objetivo secundário foi avaliar a troponina I ultrassensível (US) como preditor de mortalidade intra-hospitalar.

## Métodos

Trata-se de estudo observacional e retrospectivo, desenvolvido em UTI de hospital privado, localizado na cidade do Rio de Janeiro, Brasil, com população de indivíduos internados com diagnóstico confirmado de COVID-19 entre março e abril de 2020. A coleta de dados foi realizada por meio de consulta ao sistema eletrônico de prontuários. Foram excluídos da pesquisa os casos sem a dosagem de troponina I US e aqueles com doença renal crônica e taxa de filtração glomerular (TFG) inferior a 30 ml/min/1,73m^2^. Todos os participantes tinham idade superior a 18 anos. Este estudo foi aprovado pelo comitê de ética em pesquisa da Universidade Estadual do Rio de Janeiro. Por se tratar de estudo retrospectivo, houve dispensa do termo de consentimento livre e esclarecido. Todos os pacientes receberam terapia antimicrobiana guiada para pneumonia bacteriana comunitária no momento da admissão hospitalar, sendo o plano terapêutico ajustado conforme a evolução clínica e permitindo a reconciliação de medicamentos de uso crônico sempre que possível.

O diagnóstico de COVID-19 estava de acordo com as recomendações da OMS.^10^ Os casos foram confirmados através de técnica de reação de cadeia de polimerase (PCR) para identificação do SARS-CoV-2 em material de nasofaringe obtido por *swab* de pacientes internados em UTI. Injúria miocárdica foi definida pela presença de pelo menos um valor de troponina I cardíaca acima do limite superior da normalidade (LSN) do percentil 99, em conformidade com a Quarta Definição Universal de Infarto do Miocárdio.^11^ Foram utilizados testes de troponina I US, cujo valor de referência é inferior a 19 ng/ml. A dosagem de troponina I foi feita conforme protocolo da UTI à admissão dos pacientes ou nas seguintes condições: anormalidades globais ou regionais do movimento da parede do ventrículo esquerdo, arritmias cardíacas inexplicáveis, alterações eletrocardiográficas dinâmicas, síndrome coronariana aguda ou síndrome de insuficiência cardíaca.

As variáveis analisadas contemplaram os seguintes dados: idade, sexo, índice de massa corpórea (IMC, kg/m^2^) e as comorbidades mais prevalentes, tempo decorrido entre o início dos sintomas de COVID-19 e a internação, tempo de permanência em UTI, tempo de internação hospitalar, ocorrência de injúria miocárdica, necessidade de suporte hemodinâmico vasopressor, necessidade de suporte ventilatório invasivo, síndrome do desconforto respiratório agudo conforme os critérios de Berlim^12^ e escore SAPS 3 ( *Simplified Acute Physiology Score III* ).^13^

### Análise Estatística

As variáveis contínuas com distribuição normal foram expressas através de média e desvio-padrão e as contínuas sem distribuição normal foram expressas através de mediana e intervalo interquartil. As variáveis categóricas foram expressas em frequência absoluta e relativa. Testes de normalidade foram feitos por Kolmogorov-Smirnov. As comparações entre as variáveis contínuas foram realizadas com o teste *t* de Student não pareado ou o teste U de Mann-Whitney. Para as comparações de variáveis categóricas, utilizou-se o teste do qui-quadrado ou o exato de Fisher. Análise de regressão logística foi realizada para determinar os preditores de lesão miocárdica. As variáveis que apresentaram associação com injúria miocárdica a um nível de significância p< 0,20 foram incluídas no modelo de regressão multivariada. O método *forward stepwise* foi utilizado. A magnitude do efeito de cada variável foi estimada pelo cálculo da razão de chance (RC) e seus respectivos intervalos de confiança (IC) a 95%. A análise da curva ROC ( *Receiver Operating Characteristic* ) foi realizada para determinar o valor da troponina I US capaz de predizer mortalidade hospitalar. As funções de sobrevida foram calculadas empregando-se o estimador não paramétrico de Kaplan-Meier. Os pacientes foram estratificados por covariáveis selecionadas pelo seu provável papel prognóstico a partir de revisão da literatura. O teste log-rank foi empregado para a comparação das funções de sobrevida para cada covariável. As razões de risco (RR) foram calculadas para o prognóstico de variáveis associadas aos desfechos, com IC a 95%, segundo o modelo proporcional de Cox. Inicialmente foi realizada a análise bivariada de Cox seguida pela multivariada para os fatores com provável papel no desfecho (p<0,10). Verificou-se a proporcionalidade dos modelos de Cox pelo teste diagnóstico de resíduos de Schoenfeld. Os testes foram bicaudais e a significância estatística expressa por p<0,05. Os dados foram analisados usando o SPSS 22.0 (IBM, Chicago, IL). Os gráficos estatísticos foram gerados usando o MedCalc 19.3.

## Resultados

**Figura 1:**
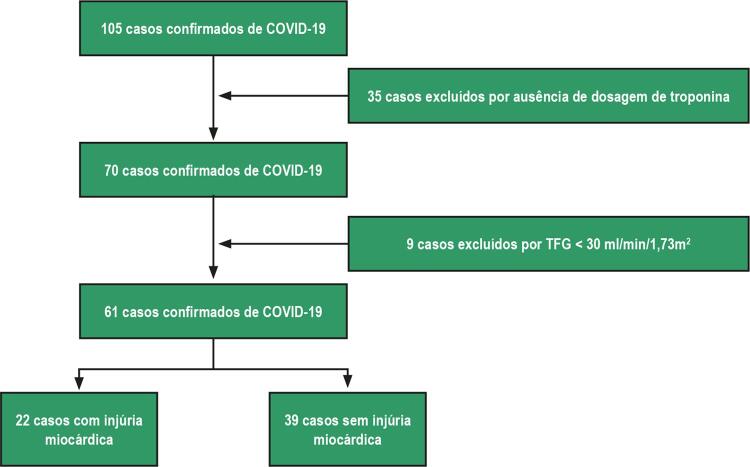
– Fluxograma de recrutamento de pacientes.

Um total de 105 casos confirmados de COVID-19 foi identificado em UTI de hospital privado, localizado na cidade do Rio de Janeiro, entre março e abril de 2020. Após serem excluídos 35 pacientes por não terem a dosagem de troponina I e 9 pacientes por apresentarem TFG < 30 ml/min/1,73m^2^, foram incluídos neste estudo 61 casos confirmados de COVID-19, dos quais, 36% possuíam injúria miocárdica ( Figura 1 ).

Desses pacientes, cerca de 63,9% pertenciam ao sexo masculino e tinham idade média de 66,1±5,5 anos. A média de tempo decorrido desde o início dos sintomas de COVID-19 e a admissão hospitalar foi de 7±6 dias, em que os tempos médios de permanência hospitalar e em UTI corresponderam a 19 e 15 dias, respectivamente. As comorbidades mais prevalentes foram hipertensão arterial (55,7%) e diabetes mellitus (27,8%), conforme descrito na Tabela 1 . Ocorreram 15 óbitos na amostra, resultando em uma taxa de mortalidade de 24,6%. Suporte intensivo invasivo foi utilizado em parcela considerável da amostra, da qual, 59% necessitou de suporte ventilatório invasivo, 57,4% fez uso de suporte hemodinâmico com vasopressores em algum momento da internação e 36% foi submetida a terapia renal substitutiva por meio de hemodiálise.

**Tabela 1:** – Características de 61 pacientes com COVID-19 internados em UTI com e sem injúria miocárdica

Características	População geral (n=61)	Com injúria miocárdica (n=22)	Sem injúria miocárdica (n=39)	p-valor
**Gerais**				
Idade	66,1±15,5	67,8±15,8	65,2±16,3	0,6010
Sexo masculino	39 (63,9%)	14 (63,6%)	25 (64,1%)	0,9710
Sintomas-internação (dias)	7±6	6±5	7±3	0,1410
Permanência em UTI (dias)	14,5 [5,2-28,7]	18,0 [8,7-33,2]	10,0 [5-28]	0,6940
Permanência hospitalar (dias)	17,0 [9,0-36]	21,5 [9,7-36,2]	13,0 [9,0-37,7]	0,5720
SAPS 3	49,7±28	55,7±27,1	46,2±32,8	0,2120
**Comorbidades**				
Hipertensão arterial sistêmica	34 (5,7%)	19 (86,4%)	15 (38,5%)	0,0001
Diabetes mellitus	17 (27,8%)	6 (27,3%)	11 (28,2%)	0,9380
DAC	4 (6,5%)	3 (13,6%)	1 (2,6%)	0,930
DPOC	2 (3,2%)	2 (9,1%)	0 (0%)	0,0560
Neoplasm	4 (6,5%)	1 (4,5%)	3 (7,7%)	0,6340
Asma	2 (3,2%)	0 (0%)	2 (3,3%)	0,2800
IMC (kg/m^2^)	29,46±6,3	32±7,6	28±5,4	0,0220
**Complicações**				
SDRA leve	2 (3,2%)	0 (0%)	2 (5,1%)	0,2800
SDRA moderada	18 (29,5%)	8 (36,4%)	10 (25,6%)	0,3780
SDRA grave	17 (27,8%)	10 (45,5%)	7 (17,9%)	0,0210
Ventilação mecânica	36 (59%)	18 (81,8%)	18 (46,2%)	0,0070
Uso de vasopressor	35 (57,4%)	18 (81,8%)	17 (43,6,%)	0,0040
Tromboembolismo venoso	11 (18%)	6 (27,3%)	5 (12,8%)	0,1590
IRA com diálise	22 (36%)	12 (54,5%)	10 (25,6%)	0,0240
Óbito	15 (24,6%)	9 (40,9%)	6 (15,4%)	0,0260

*UTI: unidade de terapia intensiva; SAPS 3: Simplified Acute Physiology Score III; DAC: doença arterial coronariana; DPOC: doença pulmonar obstrutiva crônica; IMC: índice de massa corpórea; SDRA: síndrome do desconforto respiratório agudo; IRA: insuficiência renal aguda.*

Os pacientes com injúria miocárdica tiveram tempos de internação hospitalar e de UTI discretamente superiores aos daqueles sem elevação de troponina I, porém a diferença não foi estatisticamente significativa entre os grupos. Da mesma forma, sua avaliação prognóstica através do escore SAPS 3 não diferiu significativamente, com mortalidade esperada de 55,7±27,1% em pacientes com injúria miocárdica e de 46,2±32,8% em pacientes sem injúria miocárdica (p=0,2), conforme descrito na Tabela 1 . Na regressão multivariada, os preditores de injúria miocárdica foram hipertensão arterial sistêmica (RC 9,198; IC95%: 2,246-37,665) e IMC (RC 1,143; IC95%: 1,013-1,289) conforme descrito na Tabela 2 .

**Taela 2:** – Análise multivariada de preditores de injúria miocárdica em pacientes internados em UTI

Características	RC	IC 95%	p-valor
Idade	1,010	0,977-1,045	0,543
Sexo masculino	0,980	0,330-2,907	0,971
Hipertensão arterial	10,13	2,544-40,198	0,001
DAC	6,0	0,584-61,619	0,132
IMC (Kg/m^2^)	1,108	1,009-1,218	0,033
SAPS 3	1,010	0,989-1,032	0,341

*RC: razão de chance; DAC: doença arterial coronariana; IMC: índice de massa corpórea; SAPS 3: Simplified Acute Physiology Score III.*

**Figura 2:**
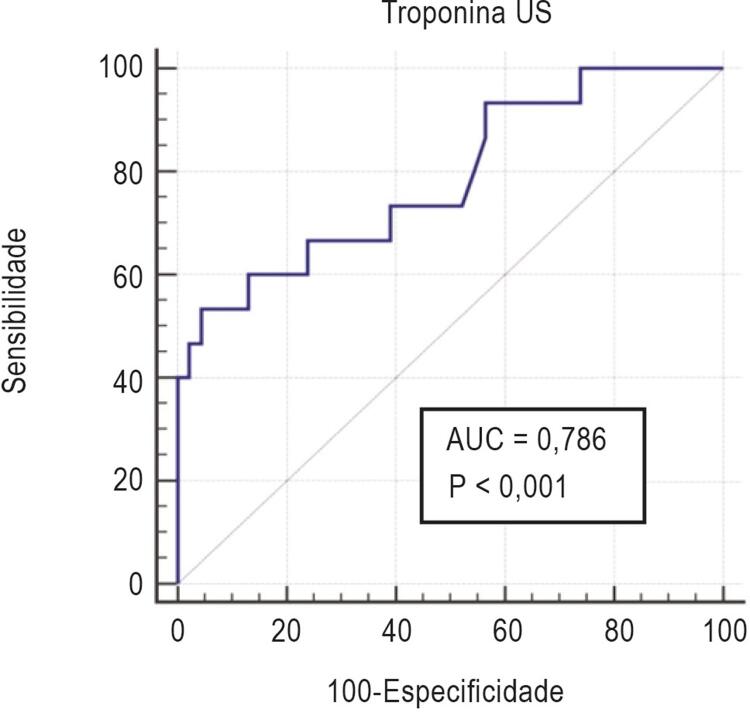
– Predição de mortalidade intra-hospitalar pela dosagem de troponina. AUC: área sob a curva ROC.

**Figura 3:**
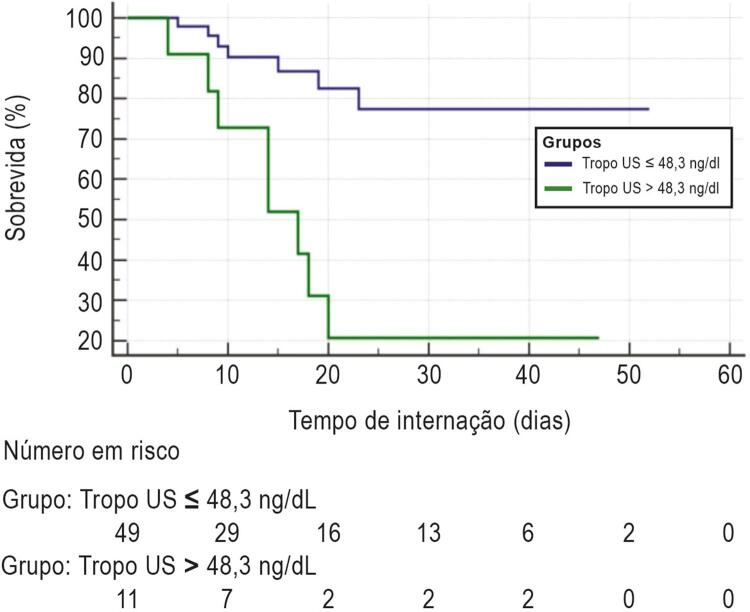
– Sobrevida de pacientes com COVID-19 internados em UTI com e sem injúria miocárdia pelo modelo de Kaplan-Meier. Tropo US: troponina ultrassensível

A análise da curva ROC foi realizada para determinar o valor da troponina I US capaz de predizer mortalidade hospitalar. Na Figura 2 , a área sob a curva ROC para o desfecho foi 0,786 (IC95%: 0,662-0,880) com p = 0,001. O valor de corte para a troponina I US foi 48,3 ng/ml. Na análise de Kaplan-Meier ( Figura 3 ), a sobrevida do grupo com troponina I US maior que 48,3 ng/ml foi 20,3 dias (IC95%: 11,4-29,2) ao passo que a sobrevida no grupo com troponina I US abaixo do valor de corte referido foi 43,5 dias (IC95%: 37,8-49,2), p = 0,0003. A análise de sobrevida bivariada de Cox estratificada pela troponina I US demonstrou relação com desfecho apenas para idade (RR = 1,046, IC95%: 1,006-1,087). Na análise multivariada, nenhuma das variáveis se mostrou preditora independente de sobrevida.

## Discussão

O termo ‘injúria miocárdica’ é empregado de forma mais abrangente para designar diferentes processos fisiopatológicos que envolvem a morte dos cardiomiócitos, podendo incluir ou não a isquemia miocárdica como causa contribuinte. Diferentes relatos mostram associação entre essa condição e a infecção por SARS-CoV-2; no entanto, o exato conhecimento sobre os mecanismos de lesão miocárdica nesses casos e sua importância prognóstica ainda representam uma lacuna de conhecimento científico a ser preenchida.^3 , 5 , 7 - 9^

As causas mais plausíveis de injúria miocárdica nos pacientes com COVID-19 incluem miocardite, hipoxemia, cardiomiopatia por estresse, *cor pulmonale* agudo e isquemia miocárdica causada por disfunção microvascular ou doença arterial coronariana epicárdica.^7 - 9 , 14^ Todavia, a contribuição isolada de cada causa à injúria miocárdica ainda não foi determinada. Os dados comprobatórios de miocardite causada por SARS-CoV-2 são escassos, por vezes faltando avaliação histológica cardíaca e análise do genoma viral, resultando em diagnóstico diferencial por suspeita clínica. A contribuição das vias de sinalização relacionadas à enzima conversora de angiotensina-2 para lesão miocárdica neste cenário também não foi adequadamente investigada.

Vem sendo postulado que a infecção pelo SARS-CoV-2 envolva intensa resposta inflamatória com um estado de hipercoagulabilidade e isquemia agravados por hipoxemia. Além disso, a resposta inflamatória sistêmica pode resultar em lesão endotelial com consequente aumento na geração de trombina e redução da fibrinólise endógena.^15^ Aspectos intrínsecos ao próprio novo coronavírus também podem contribuir diretamente para a ocorrência de injúria miocárdica, exemplificados pelos casos suspeitos de miocardite.^16 - 18^ Vários mecanismos fisiopatológicos foram propostos e podem ser resumidos nas 6 condições seguintes: disfunção endotelial, aumento do estresse oxidativo, hipoxemia, desequilíbrio entre oferta e demanda miocárdica de oxigênio, lesão miocárdica imunomediada e possivelmente lesão miocárdica direta pelo SARS-CoV-2.^18 - 20^

Embora as taxas possam variar, estima-se que até 25% dos indivíduos hospitalizados por COVID-19 necessitam de cuidados em UTI.^3 , 4^ Essas taxas variam de acordo com diferenças culturais em relação aos critérios de admissão à UTI e características regionais, como idade da população e prevalência de outras comorbidades. Da mesma forma, as taxas de letalidade em UTI variam de 22% a 67%.^21 - 24^ Em um estudo italiano com 1.591 pacientes, a mortalidade em UTI era de 26%; no entanto, uma parcela significativa de sua coorte ainda se encontrava internada em UTI no momento da publicação, o que pode ter subestimado esse indicador.^24^ Em nossa UTI, foi observada taxa de mortalidade equivalente a 24,6%, valor abaixo do esperado para a média do indicador prognóstico SAPS 3 (49,7±28%).

A incidência de injúria miocárdica em pacientes hospitalizados varia de 7% a 28%.^7 - 9^ Estudos chineses recentes apontam que pacientes com COVID-19 que necessitam de cuidados em UTI possuem maior probabilidade de evoluir com injúria miocárdica, estando essa condição associada ao maior risco de mortalidade.^5 , 25^ Nosso estudo evidenciou alta incidência de injúria miocárdica (36%) em amostra de pacientes internados em UTI brasileira com diagnóstico confirmado de COVID-19, em que a hipertensão arterial sistêmica foi um fator de risco independente da ocorrência dessa complicação. O cenário regido por esta pandemia e a necessidade de rigoroso controle de infecção hospitalar, incluindo aquela pelo novo coronavírus, limitaram o uso de métodos diagnósticos complementares, comprometendo, dessa forma, a capacidade de determinar os mecanismos de lesão miocárdica.

Uma pesquisa internacional prospectiva, realizada entre 3 e 20 de abril de 2020, com 1.216 pacientes internados por COVID-19, a maioria em UTI, buscou avaliar as principais indicações e alterações ecocardiográficas de acometimento cardíaco relacionado à infecção pelo SARS-CoV-2. As indicações mais comuns da realização do exame foram: insuficiência ventricular esquerda (40%), biomarcadores cardíacos elevados (26%) e insuficiência ventricular direita (20%). Anormalidades do ventrículo esquerdo foram relatadas em 479 pacientes (39%), sendo o comprometimento ventricular esquerdo classificado como leve (17%), moderado (12%) ou grave (9%). O referido estudo expõe o acometimento cardíaco atribuído à COVID-19, revelando considerável incidência de elevação de biomarcadores cardíacos nessa população e prejuízo à função ventricular. Todavia, nem sempre é possível determinar o exato mecanismo de injúria miocárdica.^26^

Giuseppe Lippi e Mario Plebani, em artigo publicado recentemente, revisaram 217 artigos em busca de testes laboratoriais com possível importância prognóstica para infecção pelo novo coronavírus. No entanto, 206 artigos foram excluídos por falta de informações técnicas sobre os dados apresentados. Nos 11 artigos restantes, foi possível estabelecer as principais anormalidades laboratoriais em pacientes com progressão desfavorável da COVID-19, destacando-se: aumento da contagem de glóbulos brancos, aumento da contagem de neutrófilos, diminuição da contagem de linfócitos, diminuição da albumina, aumento da desidrogenase lática, aumento da alanina aminotransferase, aumento da aspartato aminotransferase, aumento de bilirrubina total, aumento de creatinina, aumento de troponina cardíaca, aumento do dímero D, maior tempo de protrombina, aumento de procalcitonina e aumento de proteína C reativa. Com relação ao aumento de troponina I, em análise retrospectiva, foi possível identificar que aumentos superiores a 2,2 vezes o LSN correlacionam-se com resultados clínicos adversos.^27^

Estudo realizado com 2.736 pacientes com COVID-19 admitidos nos hospitais do Sistema de Saúde Mount Sinai na cidade de Nova York, entre 27 de fevereiro de 2020 e 12 de abril de 2020, observou que mesmo uma pequena quantidade de injúria miocárdica, quantificada pelo aumento da troponina, e principalmente naqueles com história de doença cardiovascular foi associada com alto rico de morte.^28^ Apesar do tamanho amostral de nosso estudo, também foi possível demonstrar com significância estatística a associação entre valores de troponina I superiores a 2,5 vezes o valor LSN e mortalidade intra-hospitalar, tendo o ponto de corte sido determinado por curva ROC. Tal informação demonstra que mesmo elevações mais modestas desse biomarcador cardíaco podem ajudar a identificar indivíduos sob risco de eventos adversos. Contudo, o uso de diferentes métodos laboratoriais comporta-se como o principal limitador da análise de um ponto de corte em grandes agregados populacionais, fazendo com que, muitas vezes, os estudos sejam em centro único. Talvez a maior riqueza na descrição metodológica dos artigos abrangendo esse tema possa facilitar o estudo por meio de meta-análise e assim melhor determinar o ponto de corte relacionado a piores desfechos clínicos.

O pequeno número de pacientes incluído nesta pesquisa e a falta de dados sobre a frequência de injúria miocárdica em pacientes assintomáticos ou apenas levemente sintomáticos infectados por SARS-CoV-2 são importantes limitações do nosso estudo. O protocolo da UTI pode ter influenciado a amostra, uma vez que após a admissão, o marcador biológico foi novamente dosado em caso de alteração do estado clínico ou de exame complementar. Outro aspecto importante foi a perda maior que 10% da amostra ocasionada pela não dosagem de troponina. Todavia, isto não foi capaz de impedir a associação de morte com aumento de troponina, mas pode ter selecionado os casos mais graves onde a troponina foi dosada, servindo os dados obtidos como parte de pesquisa exploratória em coorte retrospectiva sobre o tema. Para evitar o viés estatístico, tanto quanto possível, são necessários dados de múltiplos centros e amostras maiores para confirmar ainda mais os resultados apresentados.

## Conclusão

A incidência de injúria miocárdica de pacientes internados em UTI com diagnóstico confirmado de COVID-19 foi 36% da amostra, na qual hipertensão arterial sistêmica e IMC foram preditores de risco independentes. Foi demonstrado o impacto da injúria miocárdica na mortalidade, ao passo que a sobrevida no grupo com valor de troponina I US superior a 43,8 ng/dl foi inferior à do grupo com valores de troponina I US menores que aquele valor.
