# Supplementary material for: COVID-19 e Injúria Miocárdica em UTI Brasileira: Alta Incidência e Maior Risco de Mortalidade Intra-Hospitalar
Source: Arq Bras Cardiol. 2021 Jan 13;116(2):275–82. [Article in Portuguese] doi: 10.36660/abc.20200671 (PMC7909969; doi:10.36660/abc.20200671)
Supplement: Translation

# Translation: COVID-19 and Myocardial Injury in a Brazilian ICU: High Incidence and Higher Risk of In-Hospital Mortality

**Authors:** Jorge Henrique Paiter Nascimento, Rafael Lessa da Costa, Luiz Fernando Nogueira Simvoulidis, João Carlos de Pinho, Roberta Santos Pereira, Andrea Dornelles Porto, Eduardo Costa de Freiras Silva, Liszt Palmeira Oliveira, Max Rogerio Freitas Ramos, Gláucia Maria Moraes de Oliveira

**Affiliations:** 1 Brazil Universidade Federal do Rio de Janeiro, Rio de Janeiro, RJ - Brazil; 2 Brazil Hospital Unimed-Rio,Rio de Janeiro, RJ - Brazil

**Keywords:** COVID-19, SARS-CoV-2, Coronavirus, Betacoronavirus, Infection, Myocarditis, Myocardial Infarction, Hospitalization, Morbidity

## Abstract

**Background:**

The incidence of myocardial injury (MI) in patients with COVID-19 in Brazil and the prognostic impact of MI have not been elucidated.

**Objectives:**

To describe the incidence of MI in patients with COVID-19 in the intensive care unit (ICU) and to identify variables associated with its occurrence. The secondary objective was to assess high-sensitivity troponin I as a predictor of in-hospital mortality.

**Methods:**

Retrospective, observational study conducted between March and April 2020 with cases of confirmed COVID-19 admitted to the ICU. Numerical variables were compared by using Student t test or Mann-Whitney U test. The chi-square test was used for categorical variables. Multivariate analysis was performed with variables associated with MI and p<0.2 to determine predictors of MI. The ROC curve was used to determine the troponin value capable of predicting higher in-hospital mortality. Survival functions were estimated by use of the Kaplan-Meier method from the cut-off point indicated in the ROC curve.

**Results:**

This study assessed 61 patients (63.9% of the male sex, mean age of 66.1±15.5 years). Myocardial injury was present in 36% of the patients. Systemic arterial hypertension (HAS) [OR 1.198; 95%CI: 2.246-37.665] and body mass index (BMI) [OR 1.143; 95%CI: 1.013-1.289] were independent risk predictors. High-sensitivity troponin I >48.3 ng/mL, which was determined in the ROC curve, predicts higher in-hospital mortality [AUC 0.786; p<0.05]. Survival in the group with high-sensitivity troponin I >48.3 ng/mL was lower than that in the group with values ≤48.3 ng/dL [20.3 x 43.5 days, respectively; p<0.05].

**Conclusion:**

There was a high incidence of MI in severe COVID-19 with impact on higher in-hospital mortality. The independent risk predictors of MI were SAH and BMI. (Arq Bras Cardiol. 2020; [online].ahead print, PP.0-0)

## Introduction

The disease caused by the novel coronavirus (SARS-CoV-2, severe acute respiratory syndrome coronavirus 2) was named COVID-19 according to guidance issued by the World Health Organization (WHO). Its outbreak was first described in the city of Wuhan, China, at the end of 2019. COVID-19 was declared a public health emergency of international concern on January 30^th^, 2020, and at the time this paper was written 12 964 809 confirmed cases and 570 288 deaths had been counted worldwide.^1^By July 14^th^, 2020, Brazil had 1 926 824 confirmed cases and 74 133 deaths.^2^

Most cases of SARS-CoV-2 infection are not severe and include asymptomatic and oligosymptomatic presentations. Nevertheless, reports have suggested that up to 20% of infected individuals require hospitalization, of whom as much as 25% need admission to the intensive care unit (ICU).^3 , 4^Those rates vary according to cultural differences regarding ICU admission criteria and regional characteristics, such as population age and prevalence of other comorbidities. Development of dyspnea and severe acute respiratory syndrome are the most common indications for ICU adimission.^3 - 5^

Cardiac impairment in critically-ill patients with COVID-19 is not uncommon and comprises a wide range of presentations, such as arrhythmias, cardiomyopathies, and myocardial injury.^5 - 7^The incidence of myocardial injury in hospitalized patients varies from 7% to 28%, and correlation of myocardial injury with worse clinical outcomes has been suggested.^7 - 9^However, the causes of myocardial injury and its contribution to the prognosis are yet to be elucidated.

This study’s primary objective was to describe the incidence of myocardial injury in patients with COVID-19 admitted to the ICU and to identify possible risk factors related to its occurrence. This study’s secondary objective was to assess high-sensitivity troponin I as a predictor of in-hospital mortality.

## Methods

This is a retrospective observational study conducted in the ICU of a private hospital in the city of Rio de Janeiro, Brazil, with patients admitted with the diagnosis of confirmed COVID-19 between March and April 2020. Data were collected from the electronic medical records system. The cases without high-sensitivity troponin I measures and those with chronic renal disease and glomerular filtration rate (GFR) lower than 30 mL/min/1.73m^2^were excluded from the study. All participants were older than 18 years. This study was approved by the Ethics Committee in Research of the Rio de Janeiro State University. Written informed consent was waived because of the retrospective nature of this study. All patients received antimicrobial therapy for community-acquired bacterial pneumonia on hospital admission, and the therapeutic plan was adjusted according to clinical course, allowing medication reconciliation whenever possible.

The diagnosis of COVID-19 was in accordance with the guidance from the WHO.^10^The cases were confirmed by use of the polymerase chain reaction (PCR) to identify SARS-CoV-2 in the nasopharyngeal swab from patients admitted to the ICU. Myocardial injury was defined as the detection of at least one cardiac troponin I value above the 99^th^percentile upper reference limit (URL), in accordance with the Fourth Universal Definition of Myocardial Infarction.^11^High-sensitivity troponin I assays, whose reference value is lower than 19 ng/mL, were used. Troponin I was measured according to the ICU protocol on patient’s admission or under the following conditions: global or regional left ventricular wall motion abnormalities, inexplicable cardiac arrhythmias, dynamic electrocardiographic changes, acute coronary syndrome, or heart failure syndrome.

The following variables were analyzed: age, sex, body mass index (BMI, kg/m^2^) and the most prevalent comorbidities, time from COVID-19 symptom onset to hospitalization, length of ICU stay, length of hospital stay, myocardial injury detection, need for hemodynamic support with vasopressors, need for invasive ventilatory support, acute respiratory distress syndrome according to the Berlin definition,^12^and the Simplified Acute Physiology Score III (SAPS 3).^13^

### Statistical Analysis

The normally distributed continuous variables were expressed as mean and standard deviation, and the nonnormally distributed continuous variables were expressed as median and interquartile interval. The categorical variables were expressed as absolute and relative frequencies. The Kolmogorov-Smirnov test was used to test for normality. The continuous variables were compared by using unpaired Student *t* test or Mann-Whitney U test. The categorical variables were compared by using the chi-square test or Fisher exact test. Logistic regression was used to determine the predictors of myocardial injury. The variables that associated with myocardial injury at the significance level of p< 0.20 were included in the multivariate regression model. The forward stepwise method was used. The magnitude of the effect of each variable was estimated by calculating the odds ratio (OR) and the respective 95% confidence interval (CI). The Receiver Operating Characteristic (ROC) curve was analyzed to determine the high-sensitivity troponin I value capable of predicting in-hospital mortality. The survival functions were calculated by using the nonparametric Kaplan-Meier estimator. The patients were divided according to covariables selected by their probable prognostic role based on literature review. The log-rank test was used to compare the survival functions for each covariable. Relative risks (RR) were calculated for the prognosis of the variables associated with the outcomes, with 95% CI, according to the Cox proportional hazards model. Initially, Cox bivariate analysis was performed, and then multivariate analysis was performed for the factors possibly playing a role in the outcome (p<0.10). Schoenfeld residuals were used to check the proportional hazards of the Cox models. The tests were two-tailed, and the level of statistical significance adopted was p<0.05. Data were analyzed by use of the SPSS 22.0 (IBM, Chicago, IL). The statistical graphs were generated by using MedCalc 19.3.

## Results

This study identified 105 cases of confirmed COVID-19 in the ICU of a private hospital, in the city of Rio de Janeiro, between March and April 2020. After excluding 35 patients who had no troponin I value and 9 patients with GFR < 30 mL/min/1.73m^2^, 61 cases of confirmed COVID-19 were included in this study, 36% of which had myocardial injury ( Figure 1 ).

**Figure 1:** – Flowchart for recruiting patients.

Of the 61 patients, 63.9% were of the male sex and the mean age was 66.1±5.5 years. The mean time from COVID-19 symptom onset to hospital admission was 7±6 days, and the mean lengths of hospital and ICU stay were 19 and 15 days, respectively. The most prevalent comorbidities were arterial hypertension (55.7%) and diabetes mellitus (27.8%), as shown in Table 1 . Fifteen patients died, resulting in a mortality rate of 24.6%. Invasive intensive support was used in a significant part of the sample, 59% of which required invasive ventilatory support, 57.4% required hemodynamic support with vasopressors at some point during hospitalization, and 36% underwent renal replacement therapy by use of hemodialysis.

**Table 1:** – Characteristics of 61 patients admitted to the intensive care unit with and without myocardial injury

Characteristics	General population (n=61)	With myocardial injury (n=22)	Without myocardial injury (n=39)	p-value
**General**				
Age	66.1±15.5	67.8±15.8	65.2±16.3	0.6010
Male sex	39 (63.9%)	14 (63.6%)	25 (64.1%)	0.9710
From symptom to admission (days)	7±6	6±5	7±3	0.1410
Length of ICU stay (days)	14.5 [5.2-28.7]	18.0 [8.7-33.2]	10.0 [5-28]	0.6940
Length of hospital stay (days)	17.0 [9.0-36]	21.5 [9.7-36.2]	13.0 [9.0-37.7]	0.5720
SAPS 3	49.7±28	55.7±27.1	46.2±32.8	0.2120
Comorbidities				
Systemic arterial hypertension	34 (5.7%)	19 (86.4%)	15 (38.5%)	0.0001
Diabetes mellitus	17 (27.8%)	6 (27.3%)	11 (28.2%)	0.9380
CAD	4 (6.5%)	3 (13.6%)	1 (2.6%)	0.930
COPD	2 (3.2%)	2 (9.1%)	0 (0%)	0.0560
Neoplasm	4 (6.5%)	1 (4.5%)	3 (7.7%)	0.6340
Asthma	2 (3.2%)	0 (0%)	2 (3.3%)	0.2800
BMI (kg/m^2^)	29.46±6.3	32±7.6	28±5.4	0.0220
Complications				
Mild ARDS	2 (3.2%)	0 (0%)	2 (5.1%)	0.2800
Moderate ARDS	18 (29.5%)	8 (36.4%)	10 (25.6%)	0.3780
Severe ARDS	17 (27.8%)	10 (45.5%)	7 (17.9%)	0.0210
Mechanical ventilation	36 (59%)	18 (81.8%)	18 (46.2%)	0.0070
Vasopressor use	35 (57.4%)	18 (81.8%)	17 (43.6.%)	0.0040
Venous thromboembolism	11 (18%)	6 (27.3%)	5 (12.8%)	0.1590
ARF with dialysis	22 (36%)	12 (54.5%)	10 (25.6%)	0.0240
Death	15 (24.6%)	9 (40.9%)	6 (15.4%)	0.0260

*ICU: intensive care unit; SAPS 3: Simplified Acute Physiology Score III; CAD: coronary artery disease; COPD: chronic obstructive pulmonary disease; BMI: body mass index; ARDS: acute respiratory distress syndrome; ARF: acute renal failure.*

The patients with myocardial injury had slightly longer lengths of hospital and ICU stay than those without troponin I elevation, but the difference was not statistically significant between the groups. Similarly, their prognostic assessment by use of the SAPS 3 score did not significantly differ, with an expected mortality of 55.7±27.1% among patients with myocardial injury and of 46.2±32.8% among those without myocardial injury (p=0.2), as shown in Table 1 . On multivariate regression, the predictors of myocardial injury were systemic arterial hypertension (OR 9.198; 95%CI: 2.246-37.665) and BMI (OR 1.143; 95%CI: 1.013-1.289), as shown in Table 2 .

**Table 2:** – Multivariate analysis of myocardial injury predictors in patients admitted to the intensive care unit

Characteristics	OR	95%CI	p-value
Age	1.010	0.977-1.045	0.543
Male sex	0.980	0.330-2.907	0.971
Arterial hypertension	10.13	2.544-40.198	0.001
Coronary artery disease	6.0	0.584-61.619	0.132
Body mass index	1.108	1.009-1.218	0.033
SAPS 3	1.010	0.989-1.032	0.341

*OR: odds ratio; 95%CI: 95% confidence interval; SAPS 3: Simplified Acute Physiology Score III.*

The ROC curve was analyzed to determine the high-sensitivity troponin I value capable of predicting in-hospital mortality. In Figure 2 , the area under the ROC curve for the outcome was 0.786 (95%CI: 0.662-0.880; p = 0.001). The cut-off point for high-sensitivity troponin I was 48.3 ng/mL. In Kaplan-Meier analysis ( Figure 3 ), the survival in the group with high-sensitivity troponin I over 48.3 ng/mL was 20.3 days (95%CI: 11.4-29.2), while the survival in the group with high-sensitivity troponin I below that value was 43.5 days (95%CI: 37.8-49.2), p = 0.0003. On Cox bivariate survival analysis stratified according to high-sensitivity troponin I, only age related to the outcome (RR=1.046; 95%CI: 1.006-1.087). On multivariate analysis, no variable was an independent predictor of survival.

**Figure 2:** – Prediction of in-hospital mortality based on troponin values. AUC: area under the ROC curve.

**Figure 3:** – Survival of patients with COVID-19 admitted to the intensive care unit with and without myocardial injury, Kaplan-Meier method. HS-Tropo: high-sensitivity troponin.

## Discussion

The term ‘myocardial injury’ is widely used to designate different pathophysiological processes that involve the death of cardiomyocytes and can include myocardial ischemia as a contributing cause. Different reports have shown association between that condition and the SARS-CoV-2 infection. However, the exact mechanism of myocardial injury in such cases and its prognostic importance are yet to be known.^3 , 5 , 7 - 9^

The most plausible causes of myocardial injury in patients with COVID-19 include myocarditis, hypoxemia, stress cardiomyopathy, acute *cor pulmonale* , and myocardial ischemia caused by microvascular dysfunction or epicardial coronary artery disease.^7 - 9 , 14^However, the isolated contribution of each cause to myocardial injury is yet to be determined. The current body of evidence about myocarditis caused by SARS-CoV-2 is scarce and sometimes lacks cardiac histological assessment and viral genome analysis, resulting in differential diagnosis based on clinical suspicion. In addition, the contribution of the angiotensin-converting-enzyme II signaling pathways to myocardial damage in this scenario has not been thoroughly investigated.

It has been postulated that SARS-CoV-2 infection involves an intense inflammatory response with a hypercoagulable state and ischemia aggravated by hypoxemia. In addition, the systemic inflammatory response can result in endothelial injury with consequent increase in thrombin generation and reduction in endogenous fibrinolysis.^15^Furthermore, intrinsic aspects of the novel coronavirus can contribute directly to myocardial injury, such as the cases with suspected myocarditis.^16 - 18^Several pathophysiological mechanisms have been proposed and can be summarized in the following 6 conditions: endothelial dysfunction, increased oxidative stress, hypoxemia, imbalance between myocardial oxygen supply and demand, immune-mediated myocardial injury, and possible direct myocardial injury by SARS-CoV-2.^18 - 20^

Although the rates may vary, up to 25% of the individuals hospitalized from COVID-19 are estimated to require intensive care.^3 , 4^Those rates vary according to cultural differences regarding the ICU admission criteria and regional characteristics, such as population age and prevalence of other comorbidities. Likewise, the fatality rates in the ICU range from 22% to 67%.^21 - 24^In an Italian study with 1591 patients, the ICU mortality was 26%; however, a significant part of the cohort remained at the ICU at the time of the study publication, which might have underestimated that indicator.^24^In our ICU, the mortality rate was 24.6%, which is below the expected mean according to the prognosis indicator SAPS 3 (49.7±28%).

The incidence of myocardial injury in patients hospitalized varies from 7% to 28%.^7 - 9^Recent Chinese studies have shown that patients with COVID-19 requiring intensive care are more likely to progress with myocardial injury, which is associated with a higher mortality risk.^5 , 25^Our study evidenced a high incidence of myocardial injury (36%) in a sample of patients admitted to a Brazilian ICU with the diagnosis of confirmed COVID-19, and systemic arterial hypertension was an independent risk factor for that complication. This pandemic and the need for strict control of hospital infection, including that by the novel coronavirus, have limited the use of complementary diagnostic methods, therefore hindering the ability to determine the mechanisms of myocardial injury.

An international prospective study, conducted between April, 3, and April, 20, 2020, with 1216 patients hospitalized from COVID-19, mostly in the ICU, aimed at assessing the major indications for echocardiography and the echocardiographic changes of the SARS-CoV-2-related cardiac impairment. The most common indications for echocardiography were: left ventricular failure (40%), elevated levels of cardiac biomarkers (26%), and right ventricular failure (20%). Left ventricular abnormalities were reported in 479 patients (39%), and the left ventricular impairment was classified as mild (17%), moderate (12%) or severe (9%). That study shows the cardiac impairment attributed to COVID-19, revealing a significant incidence of elevation in cardiac biomarkers and damage to the ventricular function in that population. However, the exact mechanism of myocardial injury cannot always be determined.^26^

In a recently published study, Giuseppe Lippi and Mario Plebani have reviewed 217 articles searching for laboratory tests that might have prognostic importance for the novel coronavirus infection. However, 206 articles were excluded because of lack of technical information on the data presented. In the remaining 11 articles, the following major laboratory abnormalities in patients with unfavorable progression of COVID-19 could be established: increased white blood cell count, increased neutrophil count, reduced lymphocyte count, decrease in albumin levels, increase in lactic dehydrogenase levels, increase in alanine aminotransferase levels, increase in aspartate aminotransferase levels, increase in total bilirubin levels, increase in creatinine levels, increase in cardiac troponin levels, increase in D-dimer levels, longer prothrombin time, and increase in procalcitonin and in C-reactive protein levels. Regarding troponin I, a retrospective analysis has shown that increases greater than 2.2 times the URL correlated with adverse clinical results.^27^

A study carried out with 2736 patients with COVID-19 admitted to the Mount Sinai Health System hospitals in New York city between February 27, 2020, and April 12, 2020, has reported that even small amounts of myocardial injury, quantified by troponin elevation, mainly in patients with history of cardiovascular disease were associated with high risk of death.^28^Despite its small sample size, our study could show the statistical significance of the association between troponin I levels greater than 2.5 times the URL and in-hospital mortality, and the cut-off point was determined by use of the ROC curve. This shows that even modest elevations in that cardiac biomarker can help identify individuals at risk for adverse events. However, the use of different laboratory methods is the major limiting factor of the analysis of a cut-off point for large population clusters, with studies being conducted at single centers. The higher diversity of the methodologies of the articles on that subject might require assessment by use of meta-analysis to better determine the cut-off point related to worse clinical outcomes.

The small number of patients included in this study and the lack of data on the frequency of myocardial injury in asymptomatic or mildly symptomatic SARS-CoV-2-infected patients are important limitations. The ICU protocol may have influenced the sampling, because, after admission, the biomarker was measured again if a change in the clinical status or in complementary tests occurred. Another important aspect was the sample loss greater than 10% caused by the absence of troponin measurement. However, that could not prevent the association of death with troponin elevation, but might have selected the most severe cases, whose troponin levels were measured, and the data obtained served as part of an exploratory research in a retrospective cohort about the theme. To prevent statistical bias, data from multiple centers and larger samples are necessary to confirm the results presented.

## Conclusion

In the sample studied, the incidence of myocardial injury among patients admitted to the ICU with the diagnosis of confirmed COVID-19 was 36%, and systemic arterial hypertension and BMI were independent risk predictors. This study showed the impact of myocardial injury on mortality. In addition, survival in the group with high-sensitivity troponin I levels higher than 43.8 ng/dL was lower than that in the group with high-sensitivity troponin I levels lower than that value.
